# Effects of resveratrol treatment on bone and cartilage in obese diabetic mice

**DOI:** 10.1186/s40200-015-0141-6

**Published:** 2015-03-12

**Authors:** Joseph Cooley, Tom L Broderick, Layla Al-Nakkash, Jeffrey H Plochocki

**Affiliations:** Arizona College of Osteopathic Medicine, Midwestern University, Glendale, AZ USA; Departent of Physiology, Laboratory of Diabetes and Exercise Metabolism, Arizona College of Osteopathic Medicine, Midwestern University, Glendale, AZ USA; Departent of Physiology, Arizona College of Osteopathic Medicine, Midwestern University, Glendale, AZ USA; Department of Anatomy, Arizona College of Osteopathic Medicine, Midwestern University, Glendale, AZ USA

**Keywords:** Leptin, Resveratrol, Growth plate, Cross-sectional geometry

## Abstract

**Background:**

Resveratrol is a polyphenolic phytoalexin that has been shown to exhibit osteoprotective and chondroprotective properties. We examine the effects of resveratrol treatment on bone and cartilage tissue of obese, diabetic ob/ob mice.

**Methods:**

Eight-week-old ob/ob and lean control mice were given trans-resveratrol at an oral dose of 25 mg/kg for 3 weeks. Histomorphometric and cross-sectional-geometric variables were analyzed.

**Results:**

Ob/ob mice in our study exhibit significantly reduced femoral length, resistance to loading, and tibial growth plate total area and calcified area than lean controls (*P* < 0.05). Resveratrol treatment significantly increased cortical area in both ob/ob and control mice, but did not improve cross-sectional indicators of resistance to bending. Resveratrol treatment also reduced tibial length and calcified growth plate cartilage area in comparison to untreated mice (*P* < 0.05).

**Conclusion:**

Resveratrol treatment of ob/ob mice had mixed effects on bone histomorphometry at the femoral midshaft. Treatment increased cortical area but decreased bone length.

## Introduction

Obesity has been identified as an important risk factor in the development of disorders that affect bone health, including type 2 diabetes. According to the World Health Organization, approximately 85% of individuals diagnosed with type 2 diabetes are obese. Type 2 diabetics also have elevated rates of bone fracture, particularly at sites characterized by the presence of abundant cortical bone, such as limb bones [1-3]. The mechanism of fracture appears to be primarily structural in nature. Diabetes-related loss of cortical bone, coupled with inadequate compensatory trabecular growth, may reduce resistance to bending loads and leads to increased incidence of bone fracture [4,5].

The relationship among obesity, bone health, and diabetes is extremely complex. One significant factor in this relationship is the adipokine leptin, which is secreted in response to insulin and aids in the regulation of body mass and bone mass homeostasis [6,7]. Insulin resistance in type 2 diabetes is strongly associated with leptin levels, which in turn is associated with obesity [8,9]. Serum leptin levels have been demonstrated to be depressed in type 2 diabetics with low bone mineral density, putting them at higher risk for bone fracture [4,5]. Similarly, mice of the leptin-deficient ob/ob strain exhibit hyperglycemia and hyperinsulinemia consistent with the phenotype of type 2 diabetes and have reduced limb bone mass and length [9,10]. Leptin-deficiency in ob/ob mice also reduces bone strength via its inhibitory effect on growth plate cartilage metabolism [11]. Thus, reduced bone strength observed with type 2 diabetes may be related to the role leptin plays in the regulation of bone mass.

Resveratrol (3,5,4’-trihydroxystilbene) is a naturally-occurring phytoestrogen that has been shown to exhibit many properties that benefit health, including bone health [12]. Resveratrol selectively binds to estrogen receptors on bone and cartilage cells *in vitro* to upregulate the expression of genes that have osteoprotective and chondroprotective effects [13-15]. The interaction of resveratrol with leptin and insulin may improve insulin sensitivity in diabetics, as well as bone health [16,17]. The aim of this study is to investigate the effects of resveratrol treatment on bone and cartilage tissue of obese, diabetic ob/ob mice.

## Materials and methods

### Animals

The study utilized male mice of the strain B6.V-Lep/J (000632; Jackson Laboratories, Bar Harbor, ME). Leptin-deficient ob/ob mice of this strain are a commonly used animal model for studying diabetes and obesity. They exhibit chronically elevated glucose and insulin levels, are phenotypically similar to humans with type 2 diabetes, and demonstrate severe obesity. However, the hyperglycemia in ob/ob mice is transient, terminating between the ages of 14 and 16 weeks. To ensure the mice in our experiment were hyperglycemic, our experiment utilized mice aged 8 weeks. Lean ob/+ mice were used as controls. These heterozygous mice have the ob allele but do not exhibit the type 2 diabetes phenotype of ob/ob mice. Therefore, comparisons between these genotypes allow for clearer elucidation of the effects of resveratrol treatment while other biological factors remain similar. All mice were fed *ad libitum* and housed two per cage in a facility with a 12-hour light/dark cycle and temperature held constant at 22°C. The study was approved by the Midwestern University Institutional Animal Care and Use Committee. Care of all animals used in the study was in accordance with the recommendations in *The Guide for the Care and Use of Laboratory Animals*, National Institutes of Health, 2011.

### Experimental design

Mice were divided into groups of lean control, lean resveratrol-treated, obese control, and obese resveratrol-treated. Trans-resveratrol, the active form of resveratrol, was administered at 25 mg/kg in a volume of 0.5 to 0.75 ml once daily for 21 days via oral gavage. This dose is higher than would be attained through a normal diet has been shown to have good bioavailability following oral administration in mice [18]. Lower doses of resveratrol, such as 1 mg/kg, have been shown to have no effect on cortical bone histomorphometry [19]. The timing and duration of the treatment period coincides with the period during which ob/ob mice exhibit the type 2 diabetes phenotype. Resveratrol was mixed with a 1% solution of methylcellulose (Sigma-Aldrich, MO, USA) to form a colloid to facilitate administration. Control mice were fed with vehicle alone. During the study, there were no signs of gastrointestinal stress, changes in behavior, changes in weight, or other indicators that the treatment was not being tolerated. The specific numbers of each bone collected for each treatment group are listed in Tables 1 and 2. After three weeks of treatment, mice were sacrificed with CO_2_ asphyxiation and the hind limb bones dissected.

**Table 1:** **Two-way ANOVA of cross-sectional properties of the femoral midshaft (mean ± SE)**

	**Lean (n = 11)**	**Lean + RES (n = 12)**	**Obese (n = 14)**	**Obese + RES (n = 11)**	**Genotype Main Effect (** ***P*** **)**	**Treatment Main Effect (** ***P*** **)**	**Interaction (** ***P*** **)**
Femur length (mm)	15.4 ± 0.7	15.3 ± 1.2	13.9 ± 0.8	13.7 ± 0.9	*0.01*	0.14	0.46
Ct.Ar. (mm^2^)	2.8 ± 0.1	3.3 ± 0.2	2.8 ± 0.1	3.0 ± 0.1	*0.04*	*0.01*	0.57
I_max_ (×10^−2^ mm^4^)	10.4 ± 1.6	11.9 ± 1.7	9.2 ± 0.9	9.8 ± 1.3	*0.01*	0.43	0.73
I_min_ (×10^−2^ mm^4^)	7.2 ± 1.6	9.7 ± 2.2	5.5 ± 0.6	7.6 ± 1.1	*0.03*	0.13	0.88
J (×10^−2^ mm^4^)	17.5 ± 1.7	21.6 ± 2.2	14.7 ± 0.6	17.5 ± 0.8	*0.02*	*0.02*	0.63

**Table 2:** **Two-way ANOVA of tibia length and proximal growth plate histomorphometry (mean ± SE)**

	**Lean (n = 7)**	**Lean + RES (n = 8)**	**Obese (n = 10)**	**Obese + RES (n = 7)**	**Genotype Main Effect (** ***P*** **)**	**Treatment Main Effect (** ***P*** **)**	**Interaction (** ***P*** **)**
Tibia length (mm)	18.0 ± 0.09	17.69 ± 0.08	17.2 ± 0.07	16.92 ± 0.09	*0.01*	*0.01*	0.08
Tt.Ar (mm^2^)	0.36 ± 0.03	0.40 ± 0.02	0.36 ± 0.01	0.30 ± 0.02	*0.02*	0.89	*0.03*
B.Ar (mm^2^)	0.12 ± 0.02	0.14 ± 0.02	0.11 ± 0.01	0.09 ± 0.01	*0.04*	0.76	0.20
B.Ar/Tt.Ar (%)	33.4 ± 2.85	35.1 ± 2.05	30.8 ± 1.96	32.5 ± 1.69	*0.25*	0.44	0.98
Tt.GPl.Ar (mm^2^)	0.70 ± 0.02	0.67 ± 0.01	0.56 ± 0.01	0.37 ± 0.0	*0.05*	0.98	0.09
Md.GPl.Ar (mm^2^)	0.12 ± 0.01	0.10 ± 0.01	0.08 ± 0.03	0.05 ± 0.01	*0.01*	*0.05*	0.34
Md.GPl.Ar/Tt.GPl.Ar (%)	0.12 ± 0.01	0.10 ± 0.01	0.08 ± 0.03	0.05 ± 0.01	*0.04*	*0.04*	0.18

### Femoral midshaft cross-sectional geometry

Following sacrifice, femurs were removed, cleaned of soft tissue, and their lengths measured using digital calipers. The bones were then fixed in formalin, dehydrated in alcohol, and embedded in methyl methacrylate (Polysciences, Warrington, PA, USA). A single section approximately 150 μm in thickness was taken at the midshaft in the transverse plane of each femur using a low-speed saw (Isomet; Buehler, Lake Bluff, IL, USA). The sections were mounted on glass slides and manually ground with 600 grit abrasive paper to a thickness of 75 μm and imaged at 4x magnification with an Eclipse 55i microscope (Nikon, Inc., USA). The images were converted to black and white using the threshold function of ImageJ v1.44 and the MomentMacroJ plugin (written by M. Warfel and modified by S. Serafin) was used to calculate the cortical area (Ct.Ar.), maximum second moment of area (I_max_), minimum second moment of area (I_min_), and polar second moment of inertia (J). These geometric properties of bone in cross-section are used as surrogate indices of resistance to mechanical deformation during loading and assume bone material properties are similar among treatment groups. These surrogates are commonly used in analyses of bone strength and are derived from beam models employed by engineers [20,21]. Beam models are used to calculate mechanical characteristics from the geometric distribution of material in a section and may be applied to the analysis of structures with a length that is large relative to its width, such as limb bone.

Cortical area is indicative of resistance to compression, I_max_ and I_min_ reflect bending stiffness, and J approximates torsional stiffness.

### Proximal tibia histomorphometry

Immediately following sacrifice, tibias were harvested and their lengths measured using digital calipers. Tibias were then fixed and decalcified in solution for 4 days (Decalcifier II, Surgipath, Richmond, IL, USA), frozen in liquid nitrogen, and cryosectioned in the mid-coronal plane at a thickness of 12 μm. Sections were stained with toluidine blue and aqueous fast green (Sigma Aldrich, St. Louis, MO) and imaged at 40x magnification with an Eclipse 55i microscope (Nikon, Inc., USA).

ImageJ v1.44 was used to measure the total area of epiphyseal tissue (Tt. Ar) of the proximal epiphysis of the tibia. Tt.Ar is a measure of the total area of bone and marrow tissue in the epiphysis. Area of epiphyseal bone tissue (B.Ar) and the ratio of epiphyseal bone area to total area (B.Ar/Tt.Ar) were also collected. For all measurements of epiphyseal bone in the proximal tibia, the entire epiphysis was evaluated, not just a region of interest.

Measurements were also taken at the proximal epiphyseal growth plate of the tibia. Total area of cartilage in the growth plate (Tt.GPl.Ar), area of calcified cartilage in the growth plate (Md.GPl.Ar), and ratio of total to calcified area of the growth plate (Md.GPl.Ar/Tt.GPl.Ar) were measured. Measurements of growth plate tissue area were preferred over measurements of thickness because the growth plate borders were highly irregular, making thickness measurements difficult to reproduce reliably. The area measurements were taken for the entire growth plate, thus area of calcified cartilage is the area along the entire length of growth plate that was calcified. Bone, cartilage, and calcified cartilage were distinguished during measurement by their different staining properties. Threshold values for each staining color were used to differentiate between calcified and non-calcified cartilage tissue.

### Statistical analysis

All data are presented as means ± standard error of the mean (SE). The effects of resveratrol treatment on lean and obese mice were tested using two-way analysis of variance (ANOVA). Because bending strains are proportional to both body mass and bone length [21], body mass and femur length were used as covariates for analyses of cross-sectional geometric data. All statistics were performed using SPSS 19 software (IBM, USA). Statistical significance was set at *P* < 0.05 for all analyses.

## Results

### Resveratrol treatment significantly reduces body mass

**Figure 1:**
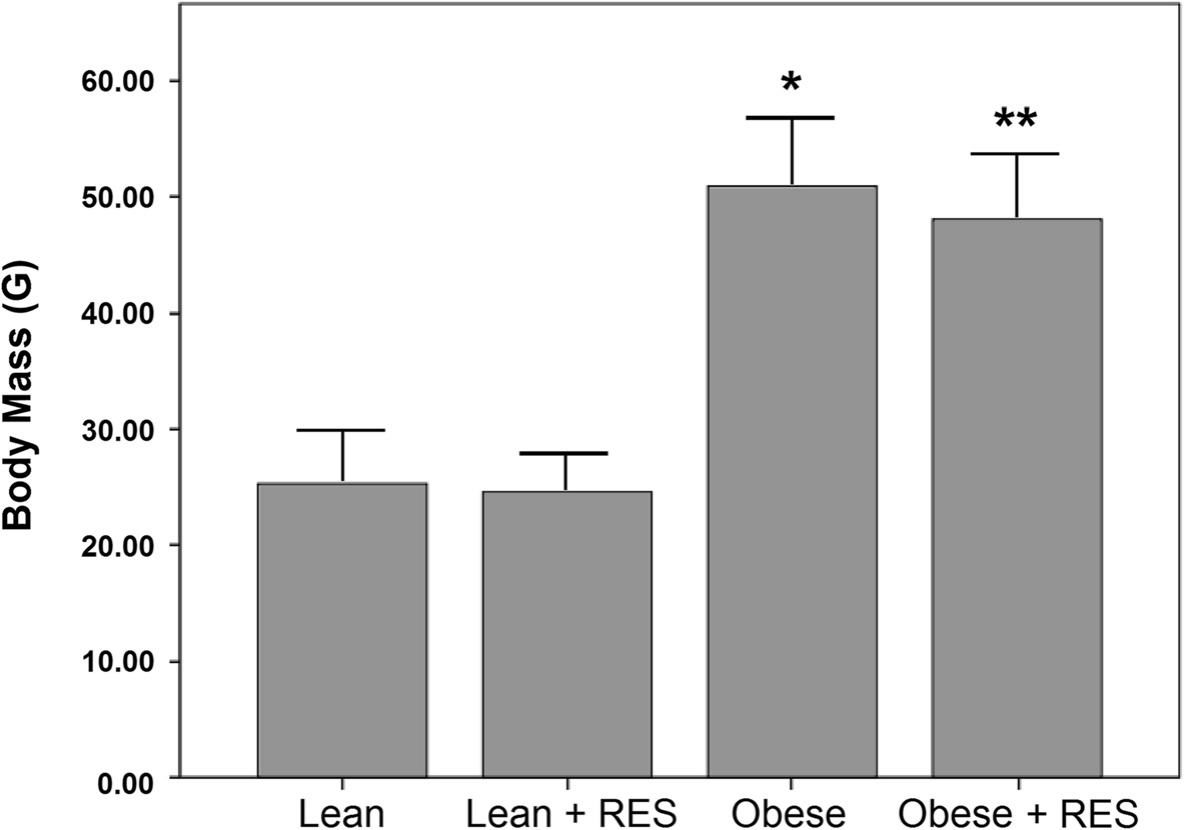
**Comparison of body mass among treatment groups.** * *P* < 0.05 lean control vs. obese control; ** *P* < 0.05 lean resveratrol-treated vs. obese resveratrol-treated.

Figure 1 shows body mass comparisons among treatment groups at the time of sacrifice. As expected, there was a genotype main effect. Lean ob/+ mice had significantly lower body mass than obese ob/ob mice (*F* = 81.5; *P* = 0.01). There was also a treatment group main effect. Mice treated with resveratrol exhibited significantly lower body mass than vehicle-treated controls (*F* = 4.4; *P* = 0.04). No interaction was found between genotype and treatment group. No differences in body mass were observed at 8, 9, or 10 weeks of age.

### Resveratrol treatment marginally improves cross-sectional geometric indicators of resistance to loading in ob/ob mice

Table 1 shows results of the two-way ANOVA for femur length and femoral midshaft cross-sectional geometric properties. Femur length was significantly reduced in obese mice in comparison to lean mice (*F* = 59.9 *P <* 0.01). No effect on femur length was found with resveratrol treatment (*P* > 0.05). Ct.Ar. and J differed significantly between obese and lean mice (*F* = 4.3; *P* = 0.04 and *F* = 5.6; *P* = 0.02 respectively), and with resveratrol treatment (*F* = 6.1; *P* = 0.01 and *F* = 5.3; *P* = 0.02 respectively). Conversely, resveratrol treatment had no effect on I_max_ and I_min_ (*P* > 0.05), but these variables differed significantly between ob/ob and ob/+ mice. Ob/ob mice exhibited reduced I_max_ and I_min_ when compared to ob/+ mice (*F* = 6.6; *P* = 0.01 and *F* = 4.9; *P* = 0.03 respectively).

### Resveratrol treatment reduces tibia length and tissue area in the proximal tibial epiphysis

Summary statistics of tibia length and histomorphometry are presented in Table 2. Obese mice had significantly shorter tibias than lean mice (*F* = 77.3; *P* < 0.01). Tibial length decreased by 4% with resveratrol treatment (*F* = 12.6; *P* = 0.01). Both Tt.Ar and B.Ar were reduced in obese mice in comparison to lean mice (*F* = 6.5; *P* = 0.02 and *F* = 4.9; *P* = 0.04 respectively), but neither was affected by resveratrol treatment (*P* > 0.05). There was also no difference in B.Ar/Tt.Ar between lean and obese mice or between resveratrol and vehicle-treated mice (*P* > 0.05).

### Resveratrol treatment decreases the area of calcified cartilage in the proximal tibia growth plate

**Figure 2:**
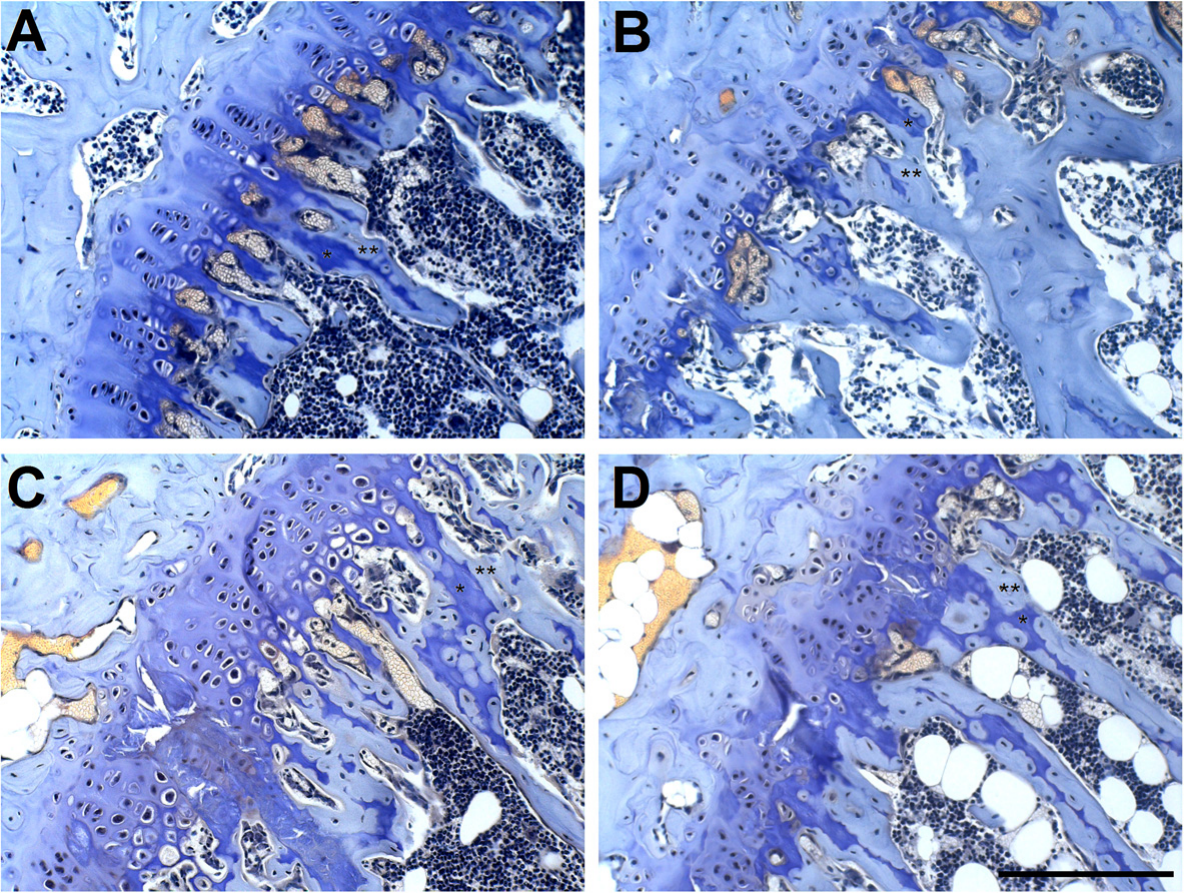
**Photo micrographs of the proximal tibial growth plate of lean control (A), lean resveratrol-treated (B), obese control (C), and obese resveratrol-treated (D) mice.** Obese mice had growth plate chondrocytes that appeared to be more disorganized than those of lean mice. Mice treated with resveratrol had significantly greater calcified cartilage areas than controls (*P* < 0.05). Toluidine blue and aqueous fast green, 20x magnification. Scale bar = 100 μm. * calcified cartilage; ** bone.

Figure 2 shows growth plate cartilage of the proximal tibia in lean and obese mice. Articular chondrocytes were much more disorganized in ob/ob mice than lean mice in both the control and resveratrol-treated groups. Table 2 depicts results of the two-way ANOVA for tibial histomorphometric comparisons. Obese mice had significantly reduced Md.GPl.Ar and Tt.GPl.Ar in comparison to lean mice (*F* = 12.7; *P* < 0.01 and *F* = 3.9; *P* = 0.05 respectively). Mice treated with resveratrol exhibited a smaller Md.GPl.Ar than controls (*F* = 4.0; *P* = 0.05), as well as a reduced Md.GPl.Ar/Tt.GPl.Ar ratio (*F* = 5.0; *P* = 0.04).

## Discussion

Leptin is an adipokine with many known functions, including the regulation of bone mass. Although early investigations into the effects of leptin on bone mass were incongruous [9,22-24], recent papers suggest leptin upregulates the expression of genes involved in ossification and bone mineralization and down-regulates bone resorption [25,26]. Consequently, ob/ob mice, which are leptin deficient, have significantly reduced bone mass and rates of bone accrual, while ob/ob mice treated with leptin regain osteogenic abilities [27]. In support of these findings, our study shows that ob/ob mice have shorter limb bones and reduced cortical area at the femoral midshaft than lean controls. Of note, ob/ob mice in our study also exhibited significantly reduced tibial growth plate cartilage area and area of growth plate calcification than lean controls, possibly because the mice in our study were still in the active skeletal growth stage of development. Leptin has been previously shown to increase chondrocyte proliferation and differentiation, reduce collagen expression by chondrocytes, and promote angiogenesis in the vicinity of hypertrophic chondrocytes [11,28,29]. These findings, combined with our own, strongly suggest leptin deficiency impairs growth of limb bones primarily by acting on growth plate cartilage.

Our study also shows femurs of ob/ob mice have significantly reduced cross-sectional geometric indices of bone strength. Femurs of ob/ob mice in our study exhibit less than half the resistance to bending and a quarter the resistance to torsion of matched controls. Similarly, ob/ob mice have previously been shown to possess significantly reduced bone mineral density, bone mineral content, and cortical thickness of limb bones due to a reduction in the rate of bone formation [9,27,30] and an increase in bone resorption [27,30]. Low serum leptin levels have been directly associated with low mineral density in type 2 diabetics, leading to high fracture rate of limb bones [4,5,31]. Fracture rates of limb bones can be as much as twice that of healthy individuals, even when controlling for other risk factors of bone fracture [32,33]. The results of our study support the hypothesis that leptin deficiency-related diabetes is associated with poor bone strength and may contribute to the high fracture rates found in obese diabetics.

Resveratrol treatment did little to improve cross-sectional geometric indices of bone strength in the mice in our study. *In vitro* studies have demonstrated that resveratrol upregulates the expression of genes that promote osteogenesis [13,14] to increase osteoblast proliferation and differentiation [34,35]. Other studies have shown that bone resorption is down-regulated by resveratrol primarily through the suppression of genes that promote osteoclastogenesis [36]. We found resveratrol treatment at a dose of 25 mg/kg does significantly increase femoral cortical area, which is an indicator of resistance to compression, and J, which is an indicator of resistance to torsion. However, resveratrol treatment did not increase resistance to bending in either ob/ob mice or lean controls. These data suggest the addition of bone that resulted in large changes in the area and shape of the bone, but only small increases along the bending axes. Because long bone fractures are primarily the result of bending loads, resveratrol treatment may not be a viable therapeutic intervention to reduce fracture rates in diabetics, although further investigation is required to determine if different doses or methods of administration may be beneficial.

Resveratrol treatment significantly reduced the area of calcified cartilage in the proximal tibial growth plate of both lean and obese mice in our study. We also found resveratrol treatment reduced tibial length in both lean and obese mice. This was contrary to what we expected. Previous studies have shown resveratrol treatment reduces chondrocyte apoptosis and the expression of matrix-degrading proteins, which suggest resveratrol exhibits chondroprotective properties [37,38]. However, resveratrol treatment has been shown to suppress the release of angiogenic factors responsible for facilitating calcification of growth plate cartilage [39]. It is possible the reduction in calcified area of the growth plate is due to the attenuating effects of resveratrol on angiogenic factors, although more research in necessary to investigate the precise mechanism. Further study is necessary to determine if there is a direct associate between resveratrol treatment and an increase in release of angiogenic factors by growth plate chondrocytes.

Overall, the results of this study suggest leptin deficiency is strongly associated with reduced limb bone length and resistance to loading in obese, diabetic ob/ob mice, which may help explain the increased fracture risk in diabetic patients. Our findings further suggest resveratrol treatment does not significantly ameliorate the negative effects of leptin deficiency. While resveratrol treatment may increase bone mass at the femoral midshaft, it does so in a manner that does not provide a significant improvement in resistance to bending, at least in the dose used in this investigation. Resveratrol may also lead to a significant decrease in bone length by inhibiting calcification of growth plate cartilage as indicated by the reduction we found in the area of calcified cartilage of the growth plate. However, more research is necessary to determine how applicable our findings are to diabetic humans given ob/ob mice exhibit the diabetic phenotype only during growth (i.e., before week 14) while most diabetic humans are adult. Additional study is also necessary to assess whether leptin deficiency adversely affects bone material properties in a manner that contributes to changes in material stiffness to increase fracture risk. It could be that a reduction in bone quality also affects bone strength.
